# Prescribing for different antibiotic classes across age groups in the Kaiser Permanente Northern California population in association with influenza incidence, 2010–2018

**DOI:** 10.1017/S0950268822001662

**Published:** 2022-10-26

**Authors:** Edward Goldstein, Bruce H. Fireman, Nicola P. Klein, Marc Lipsitch, G. Thomas Ray

**Affiliations:** 1 Center for Communicable Disease Dynamics, Department of Epidemiology, Harvard T.H. Chan School of Public Health, Boston, MA 02115 USA; 2 Kaiser Permanente Division of Research, Oakland, CA 94612 USA; 3 Kaiser Permanente Division of Research, Vaccine Study Center, Oakland, CA 94612 USA; 4 Department of Immunology and Infectious Diseases, Harvard T.H. Chan School of Public Health, Boston, MA 02115 USA

**Keywords:** Aminopenicillins, influenza, macrolides, respiratory infections, third generation cephalosporins

## Abstract

There is limited information on the volume of antibiotic prescribing that is influenza-associated, resulting from influenza infections and their complications (such as streptococcal pharyngitis). We estimated that for the Kaiser Permanente Northern California population during 2010–2018, 3.4% (2.8%–4%) of all macrolide prescriptions (fills), 2.7% (2.3%–3.2%) of all aminopenicillin prescriptions, 3.1% (2.4%–3.9%) of all 3rd generation cephalosporins prescriptions, 2.2% (1.8%–2.6%) of all protected aminopenicillin prescriptions and 1.3% (1%–1.6%) of all quinolone prescriptions were influenza-associated. The corresponding proportions were higher for select age groups, e.g. 4.3% of macrolide prescribing in ages over 50 years, 5.1% (3.3%–6.8%) of aminopenicillin prescribing in ages 5–17 years and 3.3% (1.9%–4.6%) in ages <5 years was influenza-associated. The relative contribution of influenza to antibiotic prescribing for respiratory diagnoses without a bacterial indication in ages over 5 years was higher than the corresponding relative contribution to prescribing for all diagnoses. Our results suggest a modest benefit of increasing influenza vaccination coverage for reducing prescribing for the five studied antibiotic classes, particularly for macrolides in ages over 50 years and aminopenicillins in ages <18 years, and the potential benefit of other measures to reduce unnecessary antibiotic prescribing for respiratory diagnoses with no bacterial indication, both of which may contribute to the mitigation of antimicrobial resistance.

## Introduction

Antibiotic resistance is a growing public health threat [1] that has been exacerbated during the coronavirus disease 2019 (COVID-19) pandemic [2]. Circulation of respiratory viruses results in a significant amount of antibiotic prescribing, including a substantial amount of inappropriate antibiotic prescribing for acute upper respiratory infections without a bacterial indication [3–6]. Certain antibiotic types are commonly prescribed for respiratory infections in certain age groups (e.g. macrolides in older/middle-aged adults and aminopenicillins in children), and circulation of respiratory viruses may have a significant effect on the threats of antimicrobial resistance related to those antibiotic types/age groups. While influenza infections can be a substantial source of antibiotic prescribing for acute respiratory infections (ARIs) during major influenza seasons [6], there is limited information on the contribution of influenza to annual rates of prescribing for different antibiotics in different age groups. Such information could help inform influenza vaccination efforts in different age groups and efforts at reducing inappropriate/unnecessary antibiotic prescribing for viral respiratory infections with the aim of mitigating the propagation of antimicrobial resistance for different types of antibiotics [7].

Several studies examined the contribution of influenza to overall antibiotic prescribing in children, particularly younger children. A study of antibiotic prescriptions to Scottish children aged under 5 years between 2009 and 2017 estimated that 2.4% of those prescriptions are influenza-associated [8]. Influenza infection was detected in 4.4% of acute otitis media (AOM) episodes in children in [9], and 5.3% of AOM episodes in children in [10].

A study of outpatients during two influenza seasons in the US found that antibiotic prescribing to persons with laboratory-confirmed influenza infection accounted for 17% of all antibiotic prescribing for non-pneumonia ARI [6]. However, the study [6] only refer to antibiotic prescribing for ARI, and the contribution of influenza to the overall volume of antibiotic prescribing cannot be estimated from that study. Moreover, the study [6] only refers to ARI episodes during the 4.5-month period in the 2013–2014 influenza season (driven by a novel A/H1N1 variant [11]) and the 5-month period in the 2014–2015 influenza season (a major influenza season driven by a novel A/H3N2 variant [12]) – thus, the average annual proportion of antibiotic prescribing for ARI that is influenza-associated is expected to be much lower. Our analysis of antibiotic prescribing in the Kaiser Permanente Northern California population during 2010–2018 suggested that between 2.4% and 2.7% of antibiotic prescribing in age subgroups of children aged 5–17 years, between 1.4% and 2.1% of antibiotic prescribing in age subgroups of children aged under 5 years, between 1.1% and 1.6% of antibiotic prescribing in age subgroups of persons aged over 60 years, and between 0.7% and 1.5% of antibiotic prescribing in age subgroups of persons aged 20–59 years is influenza associated [13].

While some information is available about the contribution of influenza to the volume of prescribing of all antibiotics [6, 8–10, 13], little is known about the contribution of influenza to prescribing for specific antibiotic classes in different age groups. Such information could be useful in terms of guiding influenza vaccination efforts in different age groups and in terms of reducing inappropriate antibiotic prescribing, particularly for certain commonly prescribed antibiotic types in respiratory infections (especially macrolides and aminopenicillins) in certain age groups towards addressing specific threats related to antimicrobial resistance. For example, study [14] documented a significant increase in the prevalence of resistance to extended-spectrum (ES) cephalosporins for multiple types of infections treated in US hospitals and in the incidence of hospitalisation with extended-spectrum beta-lactamase (ESBL)-producing Enterobacteriaceae, with ESBL-producing Enterobacteriaceae being listed among serious threats related to antimicrobial resistance by the US CDC [1]. For fluoroquinolones, resistance remains a significant issue related to severe outcomes for bacterial infections in the US despite recent declines in prescribing [15], and there is a substantial amount of cross-resistance for fluoroquinolones and 3rd generation cephalosporins for different organisms [16]. For aminopenicillins, high prevalence of resistance was documented in several US studies [17, 18], with amoxicillin, as well as macrolides being commonly prescribed for the treatment of community-acquired pneumonia (CAP) [19]. For macrolides, a study of *Streptococcus pneumoniae* isolates from adult ambulatory and inpatient settings at 329 US hospitals found a resistance rate of 39.5% for those isolates [20].

Using a previously developed statistical method [13, 21, 22], here we estimate influenza-associated prescribing (prescribing stemming from influenza infections and their complications) for macrolides, aminopenicillins, protected aminopenicillins (such as amoxicillin-clavulanate), quinolones and 3rd generation cephalosporins for the Kaiser Permanente Northern California population during 2010–2018. This estimation is obtained by inferring the proportion of all antibiotic prescribing that can be explained statistically by weekly variation in the incidence for the major influenza subtypes (A/H3N2, A/H1N1 and B) in a regression model that also accounts for baseline rates of prescribing not associated with influenza circulation and temporal trends. We also apply this estimation for antibiotic prescribing for respiratory diagnoses without a bacterial indication, and for ear infections in children aged under 5 years. Our estimates are relevant for evaluating the effect of influenza vaccination on antibiotic prescribing, and the mitigation of antibiotic resistance [7].

## Methods

### Data availability

Data on the circulation of influenza subtypes (A/H3N2, A/H1N1 and B) in the San Francisco Bay Area/Northern California between 2010 and 2018 are available from the California Department of Public Health, and can be accessed at https://data.chhs.ca.gov/dataset/influenza-surveillance. Proprietary data on antibiotic prescriptions (fills), related diagnoses, and influenza tests were extracted from the Kaiser Permanente Northern California's Virtual Data Warehouse and Electronic Health Records [23].

### Study population and setting

KPNC is an integrated health care system with approximately 4 million members in 2018, including approximately 3 million members between 4 and 64 years of age. KPNC members constitute more than 30% of the population in Northern California and are broadly representative of the population's racial, ethnic and socioeconomic distribution, although KPNC somewhat underrepresents those at the very lowest incomes [24, 25].

### Antibiotics and diagnoses

We extracted all antibiotic fills for macrolides, aminopenicillins, protected aminopenicillins (such as amoxicillin-clavulanate), quinolones and 3rd generation cephalosporins between September 2010 and August 2018. Antibiotic fills represented those prescriptions that were actually picked up by, or delivered to, the patient. We considered the following diagnoses categories in our analyses: (1) all diagnoses; (2) ear infections (for children aged under 18 years); (3) respiratory diagnoses without an indication of a bacterial infection – see Tables S1 and S2 in the Supplementary Material for more details on diagnoses in categories (2), (3). The reason for considering respiratory diagnoses without a bacterial indication is that antibiotic prescribing is often inappropriate for those illness episodes.

Using these data on weekly prescribing counts, as well as the data on the KPNC weekly member-time for each of the respective age groups, we calculated, for each diagnosis category, weekly rates of prescriptions (fills) for the different classes of antibiotics per 100 000 individuals in five age groups – under 5 years, 5–17 years, 18–49 years, 50–64 years, over 65 years.

### Influenza incidence

Weekly rates of respiratory samples positive (laboratory-confirmed) for influenza A and for influenza B per 100 000 Kaiser Permanente members in different age groups were used as *incidence proxies* for influenza A and B in different age groups (here, an incidence proxy is a quantity, estimated weekly, that is expected to be proportional to weekly rates of medically-attended influenza infection in those age groups). For influenza A, we further multiplied the incidence proxies for influenza A by the (weekly) proportions of influenza A specimens that were for influenza A/H1N1 and A/H3N2 in the California Department of Health data for Public Health labs [26] to define incidence proxies for influenza A/H3N2 and A/H1N1 in different age groups.

### Statistical inference

To estimate the rates of influenza-associated prescribing for different diagnosis categories, antibiotic classes and age groups, we performed separate inference for each diagnosis category, antibiotic class and each of the five selected age groups (save for the diagnosis of an ear infection, where estimates were obtained only for children aged <18 years). Adapting previously developed methodology [13, 21, 22], weekly rates of antibiotic prescribing for each diagnosis category, antibiotic class and age group were regressed linearly on the age-specific incidence proxies for the major influenza subtypes (A/H3N2, A/H1N1 and B), periodic baseline rates (with annual periodicity) of antibiotic prescribing that are not associated with influenza circulation, modelled by trigonometric (sine and cosine) functions, and temporal trend terms (quadratic polynomials in week). In the regression model, we put a lag of up to one week between influenza incidence proxies and the rates of associated antibiotic prescriptions (as described in the paragraph following eq. 1) under the assumption that there is time between influenza illness episodes and fills at Kaiser Permanente pharmacies for prescriptions for complications stemming from influenza infections.

During the 2014–2015 season, the circulating influenza A/H3N2 strains were replaced by a genetically different lineage that rendered previously used vaccines ineffective [12]. We therefore split the incidence proxy for influenza A/H3N2 into two separate proxies for the periods before and after the start of the 2014–2015 season (eq. 1). Similarly, as influenza A/H1N1 incidence was driven by novel stains starting from the 2013–2014 season [11], we split the incidence proxy for influenza A/H1N1 into two proxies for the periods before and after the start of the 2013–2014 season. The model equation for each antibiotic class/age group is as follows: let *R*(*t*) be the weekly rates of prescribing for a given diagnosis category and antibiotic class per 100 000 persons in a given age group. We linearly regress
1
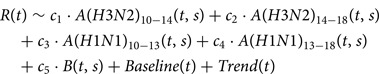
here, *A*(*H*3*N*2)_10−14_(*t*, *s*) is the incidence proxy for influenza A/H3N2 during the 2010–2014 period (so the proxy is set to zero for weeks beginning after 1 September 2014) lagged up to one week, so that *A*(*H*3*N*2)_10−14_(*t*, *s*) = *s* ⋅ *A*(*H*3*N*2)_10−14_(*t*) + (1 − *s*) ⋅ *A*(*H*3*N*2)_10−14_(*t* − 1). The number 0 ≤ *s* ≤ 1 (used in the linear interpolation for the incidence proxies on two consecutive weeks, *t* − 1 and *t*) is chosen to minimise the Akaike information criterion score of the linear regression model in eq. 1. The covariates *Baseline*(*t*) and *Trend*(*t*) are described in the 1st paragraph of this section.

Using this regression framework, we estimate the average annual rates of influenza-associated antibiotic prescribing for the five studied antibiotic classes for different diagnoses in different age groups of individuals during the 2010–2011 through the 2017–2018 influenza seasons. Influenza-attributable prescribing is estimated by the difference between the predicted value of the dependent variable (fills) with the observed levels of influenza incidence proxies to the predicted value when those proxies are set to zero. Confidence bounds for the estimates of the contribution of influenza to antibiotic prescribing are bootstrapped to account for potential residual auto-correlation as described in [21]. Additionally, we estimate the proportion of the overall prescribing for those antibiotic classes in different age groups that are influenza-associated, with proportions of antibiotic prescribing that are influenza-associated being more representative nationally than the prescribing rates themselves.

## Results

### Trends in weekly prescribing for aminopenicillins, protected aminopenicillins, 3rd generation cephalosporins, macrolides and quinolones for all diagnoses in different age groups, 2010–2018

**Fig. 1.**
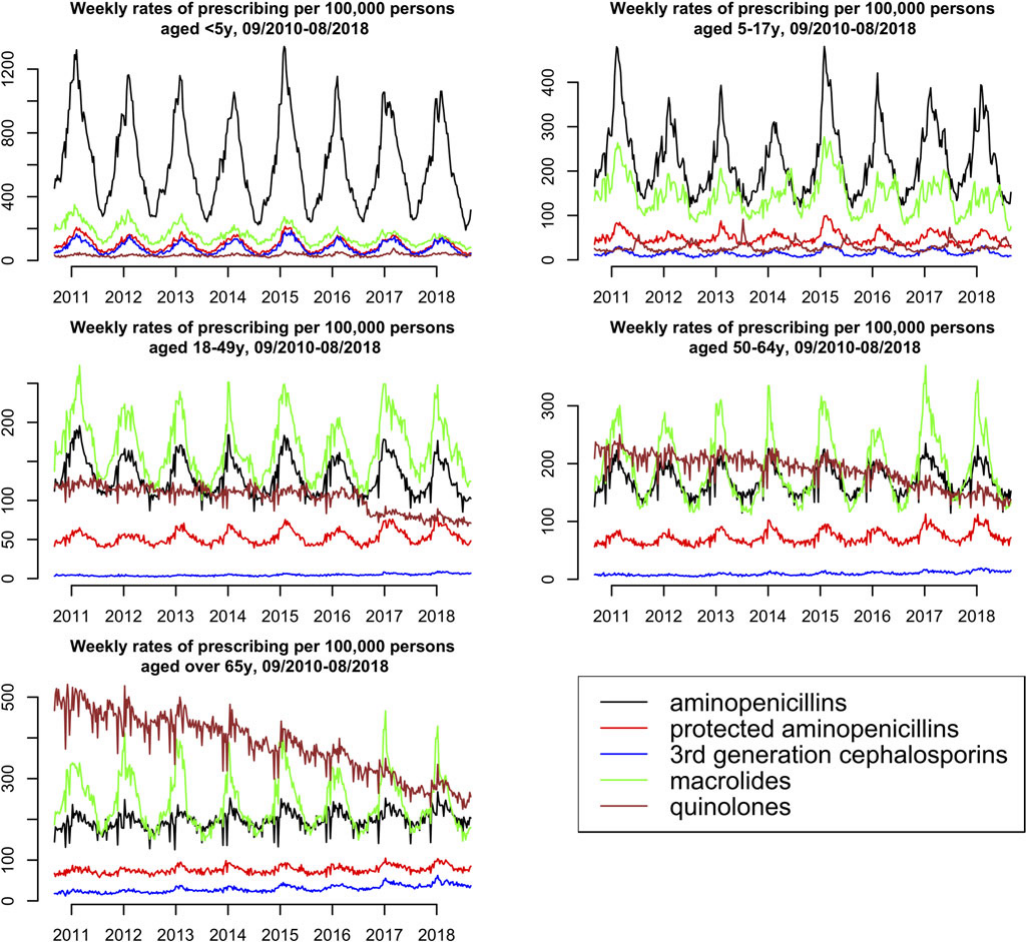
Weekly rates of prescribing for all diagnoses for aminopenicillins, protected aminopenicillins, 3rd generation cephalosporins, macrolides and quinolones per 100 000 individuals in different age groups for the Kaiser Permanente Northern California population, 2010–2018.

Weekly rates of prescribing (for all diagnoses) for aminopenicillins, protected aminopenicillins, 3rd generation cephalosporins, macrolides and quinolones per 100 000 individuals in different age groups for the Kaiser Permanente Northern California population between September 2010 and August 2018 are presented in Figure 1. For children aged under 18 years, aminopenicillins is the most commonly prescribed class of antibiotics (representing over 62% of all prescribing for the five studied classes of antibiotics in children aged under 5 years, Table 1), with pronounced seasonality (over two-fold differences between winter and summer prescribing rates) for aminopenicillin prescribing rates. For children aged 5–17 years, macrolide prescribing rates are also relatively high, exhibiting strong seasonality. For adults aged over 65 years, the highest prescribing rates are for quinolones, with rates of quinolone prescribing declining with time for all age groups of adults (see Discussion). For adults aged 18–49 years and 50–64 years, the highest prescribing rates were for macrolides, with macrolides prescribing rates also exhibiting the strongest seasonality, and with prescribing rates for aminopenicillins and protected aminopenicillins also being seasonal. Rates of prescribing for 3rd generation cephalosporins in adults are notably lower compared to the other four antibiotic classes.

**Table 1.** Overall and influenza-associated average annual rates of prescribing for macrolides, aminopenicillins, protected aminopenicillins, 3rd generation cephalosporins and quinolones per 1000 individuals in different age groups for the Kaiser Permanente Northern California population, 2010–2018

Macrolides				Aminopenicillins		
Age group	All prescribing per 1000	Flu-associated prescribing per 1000	Per cent prescribing that is flu-associated	All prescribing per 1000	Flu-associated prescribing per 1000	Per cent prescribing that is flu-associated
<5 years	83.8	1.2 (0.2–2.2)	1.4% (0.2%–2.7%)	318.6	10.4 (6.2–14.6)	3.3% (1.9%–4.6%)
5–17 years	75	2.2 (1–3.4)	2.9% (1.3%–4.6%)	117.4	6 (3.9–8)	5.1% (3.3%–6.8%)
18–49 years	87.3	2.3 (1.2–3.5)	2.7% (1.4%–4%)	67.6	1.6 (1.3–1.9)	2.4% (1.9%–2.8%)
50–64 years	100.3	4.4 (3.6–5.2)	4.4% (3.6%–5.2%)	88.3	1.2 (0.8–1.5)	1.3% (0.9%–1.7%)
65+ years	125.1	5.4 (4.4–6.3)	4.3% (3.6%–5%)	102.4	1.5 (0.9–2.1)	1.5% (0.9%–2%)
All ages	93.4	3.1 (2.6–3.7)	3.4% (2.8%–4%)	98.7	2.7 (2.2–3.1)	2.7% (2.3%–3.2%)
Third generation cephalosporins				Protected aminopenicillins		
Age group	All prescribing per 1000	Flu-associated prescribing per 1000	Per cent prescribing that is flu-associated	All prescribing per 1000	Flu-associated prescribing per 1000	Per cent prescribing that is flu-associated
<5 years	41.8	0.6 (0–1.3)	1.6% (0%–3.2%)	51.5	0.7 (0–1.3)	1.3% (0.1%–2.6%)
5–17 years	7.8	0.4 (0.2–0.6)	5.2% (2.3%–8.1%)	25.5	0.9 (0.4–1.3)	3.4% (1.4%–5.3%)
18–49 years	2.4	0.1 (0–0.1)	3% (1.7%–4.4%)	27.5	0.5 (0.4–0.7)	1.9% (1.3%–2.4%)
50–64 years	5	0.2 (0.1–0.2)	3.4% (2.3%–4.6%)	37.7	1 (0.8–1.2)	2.7% (2.1%–3.3%)
65+ years	15.4	0.5 (0.4–0.7)	3.4% (2.6%–4.4%)	39.7	0.7 (0.5–0.9)	1.7% (1.2%–2.2%)
All ages	7.9	0.2 (0.2–0.3)	3.1% (2.4%–3.9%)	32.4	0.7 (0.6–0.8)	2.2% (1.8%–2.6%)
Quinolones						
Age group	All prescribing per 1000	Flu-associated prescribing per 1000	Per cent prescribing that is flu-associated			
<5 years	17.2	0.1 (0–0.3)	0.4% (0%–1.7%)			
5–17 years	14.5	0.6 (0.1–1.1)	4.2% (0.8%–7.7%)			
18–49 years	53.9	0.5 (0.2–0.8)	1% (0.5%–1.5%)			
50–64 years	98.6	1.2 (0.8–1.6)	1.2% (0.8%–1.6%)			
65+ years	199	2.6 (1.7–3.5)	1.3% (0.9%–1.8%)			
All ages	75.9	1 (0.7–1.2)	1.3% (1%–1.6%)			

### Overall, and influenza-associated prescribing for all diagnoses

Overall annual rates of prescribing for macrolides and quinolones were highest in persons aged over 65 years (125.1 and 199 annual prescriptions correspondingly per 1000 persons), followed by persons aged 50–64 years (Table 1). Overall annual rates of prescribing for aminopenicillins, protected aminopenicillins and 3rd generation cephalosporins were highest in children aged under 5 years (318.6 annual prescriptions for aminopenicillins, 51.5 prescriptions for protected aminopenicillins and 41.8 prescriptions for 3rd generation cephalosporins per 1000 children aged <5 years).

We estimated that for the whole Kaiser Permanente population, 3.4% (2.8%–4%) of all macrolide prescriptions (fills), 2.7% (2.3%–3.2%) of all aminopenicillin prescriptions, 3.1% (2.4%–3.9%) of all 3rd generation cephalosporins prescriptions, 2.2% (1.8%–2.6%) of all protected aminopenicillin prescriptions and 1.3% (1%–1.6%) of all quinolone prescriptions were influenza-associated. Additionally, the proportion of macrolide prescribing that was influenza-associated was 4.3% (95% CI (3.6–5)) in ages over 65 years, 4.4% (3.6%–5.2%) in ages 50–64 years and between 2.7% and 2.9% in ages 18–49 years and 5–17 years. The proportion of aminopenicillin prescribing that was influenza-associated was 5.1% (3.3%–6.8%) in ages 5–17 years, 3.3% (1.9%–4.6%) in ages <5 years and 2.4% (1.9%–2.8%) in ages 18–49 years. The proportion of protected aminopenicillin prescribing that was influenza-associated was 3.4% (1.4%–5.3%) in ages 5–17 years, 2.7% (2.1%–3.3%) in ages 50–64 years and 1.9% (1.3%–2.4%) in ages 18–49 years. The proportion of 3rd generation cephalosporin prescribing that was influenza-associated was 5.2% (2.3%–8.1%) in ages 5–17 years and between 3% and 3.4% in ages 18–49 years, 50–64 years and over 65 years. For quinolones, the proportion of prescribing that was influenza-associated was 4.2% (0.8%–7.7%) in ages 5–17, and under 1.3% in other age groups. The average annual rates of influenza-associated prescriptions per 1000 persons were highest for aminopenicillins in children (10.4 (6.2–14.6) and 6 (3.9–8) prescription in ages <5 years and 5–17 years correspondingly), and for macrolides in ages over 50 years (5.4 (4.4–6.3) and 4.4 (3.6–5.2) prescriptions in ages over 65 years and 50–64 years correspondingly).

### Overall, and influenza-associated prescribing for respiratory diagnoses without a bacterial indication

Overall annual rates of prescribing of macrolides and quinolones for respiratory diagnoses without a bacterial indication were highest in persons aged over 65 years (46.4 and 16.4 annual prescriptions correspondingly per 1000 persons), followed by persons aged 50–64 years, whereas for protected aminopenicillins, annual rates of prescribing for respiratory diagnoses without a bacterial indication were highest in persons aged 50–64 years (14.9 annual prescriptions per 1000 persons), Table 2. Annual rates of prescribing for aminopenicillins and 3rd generation cephalosporins were highest in children aged under 5 years (68 and 6.8 annual prescriptions correspondingly). Influenza-associated prescriptions for respiratory diagnoses without a bacterial indication represented a high proportion of all antibiotic prescriptions to adults aged over 18 years (compare the estimates of the rates of influenza-associated prescribing in Tables 1 and 2): among influenza-associated prescriptions, between 51% and 90% of prescriptions for aminopenicillins, between 71% and 74% of prescriptions for protected aminopenicillins, between 58% and 69% of prescriptions for 3rd generation cephalosporins and between 48% and 62% of prescriptions for macrolides were for respiratory diagnoses without a bacterial indication. Additionally, the share of influenza-associated prescriptions among antibiotic prescriptions for respiratory diagnoses without a bacterial indication was higher than the share of influenza-associated prescriptions among antibiotic prescriptions for any diagnosis in persons aged over 5 years (4th column for each antibiotic class in Tables 1 *vs.* 2). For antibiotic prescriptions for respiratory diagnoses without a bacterial indication, 5.3%–6.7% of those prescriptions for macrolides in ages over 18 years, 4%–5.2% of those prescriptions for aminopenicillins in ages over 5 years, 4.9%–7.4% of those prescriptions for 3rd generation cephalosporins in ages over 18 years, 3%–4.9% of those prescriptions for protected aminopenicillins in ages over 5 years and 4.2%–5.2% of those prescriptions for quinolones in ages over 18 years were influenza-associated. The highest rates of influenza-associated prescribing for respiratory diagnoses without a bacterial indication were for macrolides in persons aged over 50 years (2.6 annual influenza-associated prescriptions per 1000 persons).

**Table 2.** Overall and influenza-associated average annual rates of prescribing for respiratory diagnoses without a bacterial indication for macrolides, aminopenicillins, protected aminopenicillins, 3rd generation cephalosporins and quinolones per 1000 individuals for the Kaiser Permanente Northern California population, 2010–2018

Macrolides				Aminopenicillins		
Age group	All prescribing per 1000	Flu-associated prescribing per 1000	Per cent prescribing that is flu-associated	All prescribing per 1000	Flu-associated prescribing per 1000	Per cent prescribing that is flu-associated
<5 years	23.6	0.4 (0–0.9)	1.6% (0%–3.6%)	68	0.7 (0–1.6)	1.1% (0%–2.3%)
5–17 years	27.9	0.9 (0–2.1)	3.2% (0%–7.4%)	43.5	1.7 (0.8–2.7)	4% (1.8%–6.2%)
18–49 years	27.6	1.5 (0.9–2)	5.3% (3.2%–7.3%)	20.5	1 (0.6–1.3)	4.6% (3%–6.3%)
50–64 years	39.1	2.6 (2.1–3.1)	6.7% (5.5%–7.9%)	20.4	1.1 (0.9–1.3)	5.2% (4.2%–6.3%)
65+ years	46.4	2.6 (1.8–3.3)	5.5% (3.9%–7.1%)	15.6	0.8 (0.6–1)	5% (3.5%–6.5%)
Ages 5 years +	33.1	1.8 (1.4–2.1)	5.4% (4.3%–6.5%)	23.6	1.1 (0.9–1.3)	4.6% (3.6%–5.6%)
Third generation cephalosporins				Third generation cephalosporins		
Age group	All prescribing per 1000	Flu-associated prescribing per 1000	Per cent prescribing that is flu-associated	All prescribing per 1000	Flu-associated prescribing per 1000	Per cent prescribing that is flu-associated
<5 years	6.8	0.1 (0–0.2)	1.2% (0%–3%)	11.4	0 (0–0.1)	0 (0%–0.9%)
5–17 years	2.6	0.1 (0–0.1)	2.1% (0%–4.7%)	11	0.3 (0–0.7)	3% (0%–6.3%)
18–49 years	0.8	0 (0–0.1)	4.9% (2.5%–7.3%)	11.7	0.4 (0.2–0.5)	3.2% (1.9%–4.4%)
50–64 years	1.8	0.1 (0.1–0.2)	6.5% (4.6%–8.4%)	14.9	0.7 (0.5–0.9)	4.9% (3.6%–6.2%)
65+ years	4.3	0.3 (0.3–0.4)	7.4% (6%–8.9%)	11.7	0.5 (0.4–0.6)	4.3% (3.1%–5.5%)
Ages 5 years +	1.9	0.1 (0.1–0.1)	5.5% (4.5%–6.5%)	12.3	0.5 (0.4–0.6)	3.8% (2.9%–4.6%)
Quinolones						
Age group	All prescribing per 1000	Flu-associated prescribing per 1000	Per cent prescribing that is flu-associated			
5–17 years	0.2	0 (0–0)	2.5% (0%–6.9%)			
18–49 years	2.8	0.1 (0.1–0.2)	5.2% (4%–6.4%)			
50–64 years	7.6	0.4 (0.3–0.5)	5.5% (4.5%–6.5%)			
65+ years	16.4	0.7 (0.5–0.8)	4.2% (3.3%–5%)			
Ages 5 years +	5.5	0.3 (0.2–0.3)	4.8% (4.2%–5.4%)			

### Overall, and influenza-associated prescribing for ear infections in children aged under 18 years

Aminopenicillins were the most commonly prescribed antibiotics for ear infections in children (198.1 and 41 such annual prescriptions in ages <5 years and 5–17 years correspondingly), with the contribution of other antibiotics to prescribing for ear infections in children being much lower. Rates of influenza-associated aminopenicillin prescribing for ear infections in children were relatively high (8.4 and 3.8 such prescriptions in ages <5 years and 5–17 years correspondingly), representing most of influenza-associated aminopenicillin prescribing in children aged under 18 years (Tables 1 and 3). The share of influenza-associated prescribing among all antibiotic prescribing for ear infections was relatively high, particularly in ages 5–17 years (e.g. 9.4% of all prescribing of aminopenicillins for ear infections, 9% of all prescribing of 3rd generation cephalosporins for ear infections and 7.2% of all prescribing of protected aminopenicillins for ear infections in ages 5–17 years were influenza-associated).

**Table 3.** Overall and influenza-associated average annual rates of prescribing for ear infection for macrolides, aminopenicillins, protected aminopenicillins, 3rd generation cephalosporins and quinolones per 1000 children aged under 18 years for the Kaiser Permanente Northern California population, 2010–2018

Macrolides				Aminopenicillins		
Age group	All prescribing per 1000	Flu-associated prescribing per 1000	Per cent prescribing that is flu-associated	All prescribing per 1000	Flu-associated prescribing per 1000	Per cent prescribing that is flu-associated
<5 years	15.6	0.6 (0.3–1)	4.2% (2.2%–6.1%)	198.1	8.4 (5.5–11.4)	4.3% (2.8%–5.7%)
5–17 years	4.6	0.2 (0.1–0.3)	4.5% (1.4%–7.6%)	41	3.8 (2.6–5.1)	9.4% (6.4%–12.4%)
All children	7.3	0.3 (0.2–0.4)	4.3% (2.5%–6.1%)	80.1	5 (3.8–6.2)	6.2% (4.7%–7.7%)
Third generation cephalosporins				Protected aminopenicillins		
Age group	All prescribing per 1000	Flu-associated prescribing per 1000	Per cent prescribing that is flu-associated	All prescribing per 1000	Flu-associated prescribing per 1000	Per cent prescribing that is flu-associated
<5 years	28.9	0.5 (0–1)	1.8% (0%–3.5%)	26.8	0.7 (0.2–1.1)	2.5% (0.8%–4.1%)
5–17 years	3.7	0.3 (0.2–0.4)	9% (6%–12.2%)	4.5	0.3 (0.2–0.5)	7.2% (4.2%–10.2%)
All children	10	0.4 (0.2–0.5)	3.8% (2.2%–5.3%)	10	0.4 (0.3–0.6)	4.1% (2.6%–5.5%)
Quinolones						
Age group	All prescribing per 1000	Flu-associated prescribing per 1000	Per cent prescribing that is flu-associated			
<5 years	6.4	0 (0–0.1)	0.5% (0%–1.8%)			
5–17 years	2.5	0.1 (0.1–0.2)	5.9% (3.8%–7.9%)			
All children	3.5	0.1 (0.1–0.2)	3.4% (2.2%–4.7%)			

## Discussion

While rates of antibiotic prescribing for ARIs, including rates of inappropriate antibiotic prescribing are fairly high [3–6], there is limited information on the contribution of influenza to antibiotic prescribing, particularly to prescribing for specific antibiotic classes/types for which antimicrobial resistance poses significant public health threats. In this study, we evaluated the contribution of influenza to antibiotic prescribing for aminopenicillins, protected aminopenicillins, third generation cephalosporins, macrolides and quinolones in different age groups of children and adults in Kaiser Permanente Northern California between 2010 and 2018. While the relative contribution of influenza to overall antibiotic prescribing is relatively small [13], here we estimated a larger relative contribution of influenza to prescribing for select antibiotics in certain age groups, e.g. over 4.3% of macrolide prescribing in ages over 50 years, 5.1% of aminopenicillin prescribing in ages 5–17 years and 3.3% in ages under 5 years, 5.2% of 3rd generation cephalosporins prescribing in ages 5–17 years and over 3% in ages over 18 years, as well as 3.4% of protected aminopenicillins prescribing in ages 5–17 years were influenza-associated. Our estimates of the relative contribution of influenza to quinolone prescribing were generally lower compared to the other studied antibiotic classes (Table 1); we also note that rates of quinolone prescribing declined with time for all age groups of adults following a series of guidelines against fluoroquinolone prescribing from the US FDA [27, 28].

The aim of this study was two-fold – informing influenza vaccination in different age groups (particularly in light of the threats of antimicrobial resistance for certain antibiotic types), and informing efforts on reducing unnecessary antibiotic prescribing, particularly prescribing of certain antibiotic types (e.g. macrolides and aminopenicillins) for respiratory diagnoses in different age groups. Unnecessary/inappropriate antibiotic prescribing for respiratory infections is an ongoing public health phenomenon [3–6]. There is substantial geographic variability in antibiotic prescribing for respiratory illness in the United States, with studies suggesting that inappropriate antibiotic prescribing for respiratory illness is most common in Southern US [29, 30]. This suggests established regional practices in addressing patients presenting with respiratory symptoms, with regional differences in antibiotic prescribing also extending to the telemedicine practice [31]. Our estimates of share of influenza-associated prescriptions among antibiotic prescriptions for respiratory diagnoses without a bacterial indication (which is higher than the share of influenza-associated prescriptions among antibiotic prescriptions for any diagnosis in persons aged over 5 years) are in support of both reducing influenza-associated antibiotic prescribing through vaccination and of reducing antibiotic prescribing for respiratory illness not involving evidence of a bacterial infection, with antibiotics being prescribed frequently and often inappropriately for such illness episodes [3–6]. The relatively high rates of influenza-associated antibiotic (particularly aminopenicillin) prescribing for ear infection in children aged under 18 years (especially children aged <5 years) further suggest the benefit of wider influenza vaccination coverage for children for reducing both antibiotic prescribing for ear infections in children and reducing the volume of illness related to ear infections, particularly AOM in younger children.

Our results may be of significance in terms guiding influenza vaccination efforts towards addressing specific threats related to antimicrobial resistance. For example, the relatively high contribution of influenza to prescribing for 3rd generation cephalosporins in persons aged over 5 years is relevant in the context of the growth in the prevalence of resistance to ES cephalosporins and the prevalence ESBL-producing Enterobacteriaceae in US hospitals [14], with ESBL-producing Enterobacteriaceae being listed among serious threats related to antimicrobial resistance by the US CDC [1]. The relatively high contribution of influenza to macrolide prescribing, particularly in persons aged over 50 years is relevant in the context of high rates of macrolide resistance in *S. pneumoniae* in the US [20]. Aminopenicillins and macrolides are frequently prescribed for the treatment of CAP [19], with high prevalence of resistance to aminopenicillins in the US documented in several studies [17, 18], and with a relatively high contribution of influenza to aminopenicillin prescribing in children, particularly school-age children (over 5% of all aminopenicillin prescribing) found in this paper. We note that antibiotic choice plays a role in outcomes for CAP, with the use of beta-lactams found to be associated with the highest rates of treatment failure in [32].

Influenza vaccination coverage in the US have increased gradually with time [33], with overall vaccination rate for the 2019–2020 season reaching 63.8%. However, those rates declined to 58.6% for the 2020–2021 season; moreover, there is substantial variability in influenza vaccination coverage rates by state [34]. Additionally, influenza vaccination coverage rates in adults aged 18–49 years are notably lower compared to other age groups [33], with vaccination rates in children aged 13–17 years and adults aged 50–64 years being quite lower compared to children aged <13 years and adults aged over 65 years. Increases in influenza vaccination coverage, particularly for certain regions in the US and certain age groups have potential to reduce the rates of influenza-related outcomes, including antibiotic prescribing. We also note that influenza vaccine effectiveness varies by age and season, with relatively low vaccine effectiveness, except for children aged under 9 years, documented during recent seasons [35] (under 40% across all age groups for the 2019–20 season; under 25% for persons aged over 9 years for the 2018–19 season; under 33% for persons aged over 9 years for the 2017–18 season). Further work is needed to assess the effect of increases in influenza vaccination coverage on the rates of prescribing for different antibiotic classes in different age groups.

Our inference method has limitations, particularly in not accounting for season-to-season variation in the circulation of respiratory viruses other than influenza, with the resulting variability in associated antibiotic prescribing. Further work is needed to assess the contribution of different respiratory viruses, including influenza to antibiotic prescribing. Prescribing patterns at Kaiser Permanente may not be broadly generalisable because KPNC frequently tests for influenza; for example, physicians elsewhere may be more likely to prescribe antibiotics for visits stemming from influenza infections (particularly undetected influenza infections). Around 22% of all antibiotic prescriptions in the KPNC database are missing a diagnosis. Though this affects the estimates of the rates of antibiotic prescribing for respiratory illness without a bacterial indication and for ear infections, this should not bias the estimates of the relative contribution of influenza to prescribing for those diagnoses.

Despite those limitations, our results provide estimates of the contribution of influenza to prescribing for five major antibiotic classes: aminopenicillins, protected aminopenicillins, third generation cephalosporins, macrolides and quinolones in different age groups of children and adults. Our results suggest a modest benefit of increasing influenza vaccination coverage for reducing antibiotic prescribing for the five studied antibiotic classes, particularly for macrolides in ages over 50 years, aminopenicillins in children aged under 18 years and 3rd generation cephalosporins in ages over 65 years, as well as the potential benefit of other measures to reduce unnecessary antibiotic prescribing for respiratory diagnoses with no bacterial indication in persons aged over 5 years, both of which may further contribute to the mitigation of antimicrobial resistance.

## Supplementary material

For supplementary material accompanying this paper visit http://doi.org/10.1017/S0950268822001662. click here to view supplementary material
